# Suppression of aquaporin, a mediator of water channel control in the carcinogenic liver fluke, *Opisthorchis viverrini*

**DOI:** 10.1186/1756-3305-7-224

**Published:** 2014-05-14

**Authors:** Sirikanda Thanasuwan, Supawadee Piratae, Paul J Brindley, Alex Loukas, Sasithorn Kaewkes, Thewarach Laha

**Affiliations:** 1 Department of Parasitology, Faculty of Medicine, Khon Kaen University, Khon Kaen 40002, Thailand; 2 Department of Microbiology, Immunology and Tropical Medicine, Research Center for Neglected Diseases of Poverty, School of Medicine & Health Sciences, George Washington University, Washington, DC 20037, USA; 3 Centre for Biodiscovery and Molecular Development of Therapeutics, Australian Institute of Tropical Health and Medicine, James Cook University, Cairns, Queensland 4878, Australia; 4 Liver Fluke and Cholangiocarcinoma Research Center, Faculty of Medicine, Khon Kaen University, Khon Kaen 40002, Thailand

**Keywords:** *O. viverrini*, Cholangiocarcinoma, RNA interference, Aquaporin, Liver fluke, Tegument

## Abstract

**Background:**

Opisthorchiasis and *Opisthorchis viverrini*-associated bile duct cancer represent major public health threats in Thailand and Laos. The tegument of this food borne fluke plays pivotal roles in parasite metabolism, homeostasis and osmoregulation. Excretory/secretory products also pass from the fluke to the biliary environment, products that likely underlie pathogenesis of liver fluke infection. Aquaporins (AQPs), belong to the major intrinsic protein superfamily of integral plasma membrane channel proteins that selectively transport water across cell membranes. AQPs play key roles as water and ion transport channels through the tegument of helminth parasites.

**Methods:**

Here, two forms of AQP mRNAs from the adult developmental stage of *O. viverrini*, termed *O. viverrini* aquaporin-1 and -2 (*Ov-aqp-1* and -*2*) were investigated. Roles of *Ov-aqp-1* and -*2* in the movement of water across the tegument of this carcinogenic liver fluke were investigated using RNA interference.

**Results:**

*Ov*-AQP-1 and *Ov*-AQP-2 contain unique characteristic asparagine-proline-alanine (NPA) motifs of AQP transmembrane proteins. Phylogenetic analysis indicated that *Ov*-AQPs belong to an expanding group of aquaglyceroporin-like water channel proteins characterized from helminth and protozoan parasites, which is pivotal to the specialized requirements of water and solute control during parasitism. Elevated transcription of *Ov-aqp-1* was evident in the egg, cercaria, metacercaria and adult stages of *O. viverrini,* whereas *Ov*-*aqp*-2 transcripts were detected at higher level in egg, metacercaria, cercaria and adult stage, respectively. RNA interference using electroporated dsRNA suppressed transcript levels of *Ov-aqp-1* and *Ov-aqp-2* in adult worms by 58-99% over periods of up to 16 days *in vitro*. Suppression of *Ov-aqp-1* and *Ov-aqp-2 in vitro* disabled water transport in adult flukes.

**Conclusion:**

The apparently pivotal roles of *Ov*-AQP in solute homeostasis at the fluke surface suggest that deeper investigation will be informative for the pathophysiology of *O. viverrini*, and may uncover intervention targets, particularly in view of the singularly notable predilection of this pathogen for residence within ducts of the biliary tree.

## Background

*Opisthorchis viverrini* is highly endemic in Southeast Asia, where more than 10 million people are infected [1,2]. Studies with hamster models of both chronic *O. viverrini* infection and exposure to nitrosamines have determined that host responses are key factors in carcinogenesis of liver fluke-induced cholangiocarcinoma [3]. Membrane proteins of *O. viverrini* without orthologues/paralogues in mammals are of particular interest for the development of vaccines and drugs, because the absence of host homologues raises therapeutic margins of safety [4-6].

AQPs are the major intrinsic protein (MIP) of integral plasma membrane channel proteins that are passively permeated by water and small, uncharged solutes [7,8]. AQPs have been identified based on their highly conserved dual asparagine-proline-alanine (NPA) boxes, critical for the formation of a water-permeating pore. The sequences of conventional AQPs have only minimal or modest identity to one another but they share conserved six transmembrane domains and hydrophobic NPA box-like repeats [9,10]. Moreover, some AQP-like sequences exhibit only poor sequence conservation for the NPA motifs [11]. The NPA motifs of AQPs play crucial roles for movement of water across cell membranes [8].

AQPs have been investigated in several parasites and observed to provide key functions in transport of water and other small solutes. Moreover, AQPs facilitate and inhibit uptake of lactate and other anthelmintics [12-14]. In schistosomes, AQP is a major tegument protein with functions in trans-tegumental water movement, absorption of nutrients and other metabolites [14,15]. AQPs are highly expressed in the transcriptomes and tegumental proteome of *O. viverrini *[16,17]. In this study, discrete mRNA sequences encoding two AQP-like transporters were isolated from a cDNA library established from the adult stage of *O. viverrini*. The properties of these water channel transporters were investigated using bioinformatics approaches, phylogenetic analyses and gene silencing via RNA interference to highlight the physiological roles of AQPs in liver fluke biology and parasitism.

## Methods

### *Opisthorchis viverrini*

Metacercariae of *O. viverrini* were collected from the flesh of naturally infected cyprinid fish from Khon Kaen province, Thailand by digestion with 0.25% pepsin, as described [18]. Syrian golden hamsters, *Mesocricetus auratus*, purchased from the Animal Unit, Faculty of Medicine, Khon Kaen University were infected with 50 metacercariae by orogastric gavage [19]. Hamsters were maintained at the animal facility, Faculty of Medicine, Khon Kaen University using protocols approved for animal experimentation by the Animal Ethics Committee of Khon Kaen University, based on the Ethics of Animal Experimentation of the National Research Council of Thailand (Approval number AEKKU43/2555). Hamsters were euthanized six weeks after infection, when adult flukes were recovered from bile ducts and the gall bladder. The worms were rinsed in sterile 0.9% NaCl to remove residual host cells and debris.

Eggs of *O. viverrini* were collected from worms recovered from euthanized hamsters; flukes were maintained in RPMI media containing antibiotics (streptomycin/penicillin, 100 μg/ml) at 37°C, in an atmosphere of 5% CO_2_ and incubation for 18 h [20]. Eggs were collected by centrifugation at 5,241 *g* for 10 min and stored at -70°C. Cercariae of *O. viverrini* were shed from naturally infected *Bithynia* sp. snails collected in farmlands in Khon Kaen province [21]. Snails were placed into plastic containers filled with de-chlorinated water, 4–5 snails per container, and exposed to the light for 2 h, after which cercariae were collected by centrifugation of the supernatant water at 5,241 *g*, 10 min.

### Isolation of aquaporin genes from *O. viverrini*

cDNA sequences encoding full open reading frames (ORFs) of *Ov-aqp-1* and *Ov-aqp-2* were amplified by PCR from a cDNA library of transcripts from the adult developmental stage of the fluke [16]. The specific primers for PCR amplification of the *Ov*-*aqp* genes were designed based on expressed sequence tags (ESTs) and EST contigs. cDNA sequences encoding full length ORFs of *Ov-aqp-1* (GenBank accession EL618688) [16] and *Ov-aqp-2* (OV_contiq1681, available at http://bioinfosecond.vet.unimelb.edu.au/) [22] were identified from our previous transcriptomics study. The primers for Ov-AQP-1 F were 5′-AGCATGGCTGGTAGTCTCTCATC and Ov-AQP-1R5′-AGCGGATCCTCAGTTTTTTTTCTGGCG. The primers of *Ov*-AQP-2 F were 5′-AGCCATATGATGAGTTTGGAATGCGAAAC and *Ov*-AQP-2R5′-AGCGGATCCCTAGGCAAGCAGTTCAGTTC. PCR reaction mixes included the *O. viverrini* cDNA library (100 ng), 0.2 mM dNTP, 1.5 mM MgCl_2_ with 1 unit *Taq* polymerase (Invitrogen, USA). Amplification was accomplished with 35 cycles of denaturation at 95°C for 1 min, annealing at 60°C for 1 min, extension at 72°C for 2 min and a final extension at 72°C for 10 min. The amplicons were separated and sized by electrophoresis through agarose followed by staining with ethidium bromide. Products of interest were isolated from the gel using a kit (GeneJET™ gel extraction, Fermentas, EU), ligated into the pGEM-T Easy vector (Promega, USA), after which ligation products were used to transform *Escherichia coli* strain JM109 competent cells (Promega). Plasmids isolated from the resulting colonies were sequenced using BigDye terminator method (1^st^ BASE, Singapore); sequences were analyzed using Blast search against GenBank databases [23] and compared to consensus sequences of *Ov*-AQPs from ESTs of *O. viverrini *[16,22]. Recombinant plasmids containing cDNA sequences encoding the entire ORFs of *Ov-aqp-1* and *Ov-aqp-2* were termed *pOv-aqp-1* and *pOv-aqp-2.*

### Sequence and phylogenetic analyses

Nucleotide sequences and chromatograms were evaluated using BioEdit V7.0.5 [24]. The edited sequences were translated to ORFs with assistance of software at http://bio.lundberg.gu.se/edu/translat.html. Signal peptides from the deduced amino acid sequences were predicted and analyzed by SignalP 3.0 Server at http://www.cbs.dtu.dk/services/SignalP/. Transmembrane helices were predicted by using TMHMM Server v. 2.0 (http://www.cbs.dtu.dk/services/TMHMM-2.0/). N-Glycosylation sites were predicted by using the NetNGlyc 1.0 Server at http://www.cbs.dtu.dk/services/NetNGlyc/. Other divergent sequences were compared and multiple alignments constructed by ClustalW in the BioEdit program [24]. ORFs of *Ov*-AQPs were aligned with well characterized AQP protein sequences from informative species using ClustalW in the BioEdit program [24]. A phylogenetic tree was constructed with p-distance matrix using the neighbor-joining method [25] with 1,000 bootstrap samplings in the MEGA version 6.06 [26].

### RNA extraction and quantitative real time reverse transcription PCR

Real time, reverse transcription PCR was undertaken to monitor the expression of the *Ov-aqp* genes during the developmental cycle of the liver fluke - egg, cercaria, metacercaria and adult stages were examined, and at intervals after exposure by electroporation to dsRNA. Briefly, total RNA was extracted from the developmental stages of the parasite using TRIZOL (Invitrogen). Any residual DNA remaining in the RNA preparations was removed by DNase digestion. Double stranded cDNA was synthesized from equal amounts of total RNA template (1 μg) using a cDNA synthesis kit (Fermentas). Quantitative real-time PCR was performed using custom SYBR Green Assays. The primers to detect *Ov-aqp*-*1* (spanning coding DNA positions 3–280), were AQP1_EXF, 5′-GGCTGGTAGTCTCTCATC-3′ and AQP1_EXR, 5′-CGTATCCCATAGTACCGCTG-3′. *Ov-aqp-2* transcripts (spanning nt positions 16 to 256) were designated Ov-AQP2_EXF: 5′-GAAACCCGATTTCGAAGAGG and Ov-AQP2_EXR: 5′-TGATCCCGGAGAAGAATACG. PCRs were performed in triplicate using SYBR Green reagents and a thermal cycler with a real time detector (ABI 7500); SYBR Green reactions were prepared by adding 12.5 μl of SYBR Green Master Mix (TAKARA Perfect Real-time Kit, Japan), 0.5 μl (10 mM) of forward primer and reverse primers, 0.5 μl of reference dye (ROX), 1 μl (equivalent to 50 ng of total RNA) of first-stand cDNA and water to a final volume of 25 μl. The thermal cycling conditions used were: initiation pre-heat for one cycle at 95°C, 10 min; 40 cycles of denaturation at 95°C, 30 sec; annealing at 55°C, 30 sec; extension at 72°C, 45 sec. Expression levels of the *Ov-aqp-1*, *Ov-aqp-2*and *actin* mRNAs (OvAE1657, GenBank EL620339) were determined as described [20].

To determine the extent of gene silencing induced by dsRNAs, the mRNA expression levels of *Ov-aqp-1* and *Ov-aqp-2* (or firefly luciferase as an irrelevant control, below) were normalized with *actin* mRNA and presented as the unit value of 2^-ΔΔCt^ where ΔΔCt = ΔCt (treated worms) - ΔCt (non-treated worms) [20,27]. Data are presented as the mean ± 1 standard deviation. Differences between groups were assessed using Student’s *t*-test (GraphPad Prism Software); *p* values of ≤ 0.05 were considered statistically significant.

### Preparation and delivery of dsRNA

dsRNAs were designed to span 605 nt of *Ov-aqp-1* (full ORF spanning nt193-798) and 575 nt of *Ov-aqp-2* (full ORF spanning nt 201–776). Target sequences were amplified from plasmids *pOv-aqp-1* and *pOv-aqp-2* (above) using primers flanked with a T7 RNA polymerase promoter sequence, indicated in underlined italic bold faced, at the 5′ end. *Ov-aqp1* was generated using primers ds-aqp1_T7-F, 5′-***TAATACGACTCACTATAGGG***GGTAGCAACGTCTCGGCTand ds-aqp1_T7-R, 5′-***TAATACGACTCACTATAGGG***GTAGAGTAACACTCCGAG. *Ov-aqp-2* was generated using primers ds-aqp2_T7-F, 5′***-TAATACGACTCACTATAGGG***AGTGAGTCTTGGCTGGGGTA and ds-aqp2_T7-R, 5′-***TAATACGACTCACTATAGGG***TACGGTCCGACGATTGGTAT. dsRNAs were synthesized using a MEGAscript RNAi Kit (Ambion, USA). The irrelevant negative control, *luciferase* (LUC) dsRNA was constructed from plasmid pGL3-basic (Promega), because this sequence does not match any targets in the *O. viverrini* genome. It was amplified using primers ds-LUC_T7-F5′-***TAATACGACTCACTATAGGG***TGCGCCCGCGAACGACATTTA and ds-LUC_T7-R5′-***TAATACGACTCACTATAGGG***GCAACCGCTTCCCCGACTTCCTTA [28]. The thermal cycling conditions were 35 cycles of denaturation at 94°C, 30 sec; annealing at 55°C, 30 sec; and extension at 72°C, 60 sec. Amplicons were sized by electrophoresis and purified, as above. Concentrations of dsRNA were determined by spectrophotometer (NanoVue, GE Healthcare, USA).

To deliver *Ov-aqp* dsRNAs, adult worms (30 per treatment group) were washed with sterile phosphate buffer, then transferred to a cuvette, 4 mm gap (Bio-Rad) in 100 μl electroporation buffer (RPMI-1640, 1x antibiotic/antimycotic, 1% glucose, 1 mM *trans*-epoxysuccinyl-L-leucylamido (4-guanidino) butane (E64) (Sigma) containing 50 μg *Ov-aqp*-1, *Ov-aqp*-2 or *luc* dsRNAs. Each group of worms was subjected to square wave electroporation using a single 20 ms impulse at 125 V (Gene PulserXcell, Bio-Rad, USA). Subsequently, worms were maintained in RPMI culture medium (one ml culture medium in each well of a 24 well plate) supplemented with 2 mg/ml of dsRNA at 37°C under 5% CO_2_ in air. Similarly treated worms in electroporation buffer without dsRNA served as controls. Worms were soaked in 2 mg dsRNA for 16 days (*Ov-aqp*-1) and 7 days (*Ov-aqp*-2) with changes of media containing dsRNA every second day. Worms were collected for analysis on days 1, 3, 6, 10 and 16 for *Ov-aqp-1* and on days 1, 3 and 7 for *Ov-aqp-2* following electroporation.

### Water transport assay and size measurements of parasites

Water transportation in normal worms was investigated to obtain determine optimal times for observation of the transformed worms. The volume of water transport via the body wall of adult flukes was investigated by monitoring swelling of the flukes after exposure to water. Wild type adults were incubated in distilled water for 5, 10, 30, 60 and 120 min. Photomicrographs were captured on these cultures, after which surface area of the flukes was measured using the NIS-Elements D 3.22.00 software (Build 710) – Driver selection (Nikon, Japan). The function of the *O. viverrini* AQPs as water channels was investigated using RNAi to knock down the genes after treatment with *Ov*-*aqp*-*1*, *Ov*-*aqp*-*2* and *Ov*-*aqp*-*1* + *Ov*-*aqp*-*2* dsRNAs. Water transportation through the tegument of control and dsRNA-treated worms was determined by measuring the surface area of the fluke after placing the worms in water for 10 min. Briefly, worms electroporated with dsRNAs of *Ov-aqp* or *luc* were cultured in complete RPMI medium for 24 h prior to transfer to hypo-osmotic medium (distilled water) for 10 min. Photomicrographs were recorded and the surface area of individual worms ascertained before and after exposure to the hypo-osmotic conditions. To compare surface area of the liver flukes before and after transfer, images were recorded so that the surface area of individual adult worms could be determined. The liver flukes were photographed before and 10 min after transfer into water, and the sizes of a sample of individual parasites from control and *Ov*-*aqp* suppressed groups were ascertained and compared.

### Histological examination

To observe swelling of the worms, sections of fixed flukes after dsRNA treatment for 1, 3 and 7 days were examined. Briefly, the whole worms were fixed in hot 10% buffered formalin overnight then processed conventionally. Adult worms in paraffin sections were de-paraffinized in xylene three times, five minutes each. The 4 μm thick sections were stained with hematoxylin and eosin. Thereafter, the sections were dehydrated, cleared and mounted. Histology of adult worms was compared and contrasted using light microscopy [29].

### Statistical analysis

Means ± standard deviations were calculated and presented using Microsoft Excel or GraphPad Prism software. Differences between groups were assessed using Student’s *t*-test (GraphPad Prism Software, USA); *p* ≤ 0.05 was considered statistically significant.

## Results

### Sequences and structure of *O. viverrini* aquaporin

**Figure 1:**
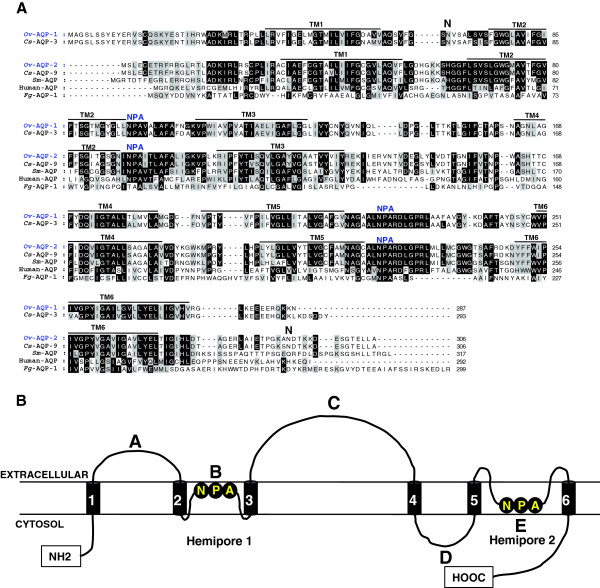
**Deduced amino acid sequences, multiple sequence alignment, and predicted secondary structure of aquaporins from *Opisthorchis viverrini*.** Multiple sequence alignment of aquaporins from *Opisthorchis viverrini* and other eukaryotic organisms **(panel A)**. Homologous protein sequences are derived from GenBank: *Ov-*AQP-1 (KF697690), *Ov-*AQP-2 (KF697691), *Cs*-AQP-3 and *Cs*-AQP-9 from *Clonorchis sinensis* (GAA31414 and GAA55320), *Sm*-AQP from *Schistosoma mansoni* (XP_002572046), humanAQP from *Homo sapiens* (CAG46822) and *Fg*-AQP-1 from *Fasciola gigantica* (AD032834). Schematic representation of the predicted architecture of *Ov*-AQPs in the cell membrane **(panel B)**. *Ov*-AQPs consist of 2 hemipores, each hemipore containing 3 transmembrane domains. Hemipore-1 is composed of transmembrane domains 1, 2, 3 and hemipore-2 is composed of transmembrane domains 4, 5, and 6. The pore-forming signature motif NPA (Asn-Pro-Ala) is located in both loops B and E.

The full length cDNA sequences encoding two isoforms of *O. viverrini* AQPs (*Ov*-AQPs) were designated *Ov-aqp-1*and *Ov-aqp-2*; nucleotide sequences of *Ov-aqp-1* and *Ov-aqp-2* have been assigned GenBank accession numbers KF697690 and KF697691, respectively. *Ov*-*aqp-1*and *Ov*-*aqp-2* are 864 and 921 nt and encode 287 and 306 amino acid ORFs, respectively. Both *Ov-*AQP-1 and *Ov*-AQP-2 are predicted to contain six- transmembrane domains (TM1-6) connected by five loops (A-E) (Figure 1). Potential *N*-glycosylation sites are present at Asn-67 in *Ov*-AQP-1 and Asn-293 in *Ov*-AQP-2 (Figure 1A). *Ov-*AQP-1 and *Ov-*AQP-2 sequences contain conserved NPA motifs as found in other AQPs. Both the amino and carboxyl termini of *Ov*-AQP-1 and *Ov-*AQP-2 are predicted to be intracellular *Ov-*AQP-1 consists of two hemipores, each hemipore containing three transmembrane domains (Figure 1B). The pore that forms in the AQPs is composed of two hemipores where loops B and E form the pore-forming signature motif, NPA (Asn-Pro-Ala) via an overlap to form the characteristic pore between the membrane bilayer (Figure 1B). It is noteworthy that both of these two new water channel molecules include the aspartic acid residue (D) in the second NPA box that is a diagnostic sequence for aquaglyceroporins, a category of aquaporin-like water channel proteins discrete from the ‘classical’ aquaporins. Aquaporins *sensu stricto* are water selective or specific water channels, whereas the aquaglyceroporins are permeable to other small, uncharged molecules, in particular glycerol, in addition to water (Benga, 2012).

### Phylogenetic analysis of *Ov*-AQP

**Figure 2:**
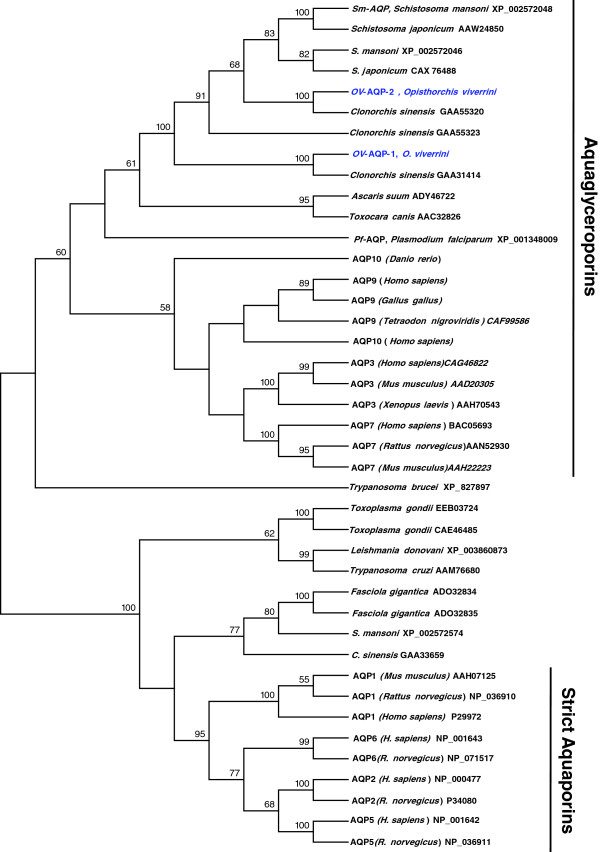
**Phylogram constructed using the neighbor joining method to compare the relationship between the open reading frames of *Ov-*AQPs of *Opisthorchis viverrini *and homologues within the aquaporin superfamily*.*** The accession numbers for sequences included in the phylogenetic analysis are shown in each operational taxonomy unit. The numbers above the branches refer to bootstrap values (where greater than 50% support was obtained). Note the separate branches for strict aquaporins and aquaglyceroporins. The novel *O. viverrini* aquaporins belong to the latter assemblage, indicating that they can transport additional permants beyond water.

The phylogenetic relationships of *Ov-*AQP-1 and *Ov-*AQP-2 protein sequences were investigated by comparison with well-characterized AQPs from various animals [9]. A neighbor-joining tree indicated that *Ov*-AQPs formed a clade with AQPs from several invertebrates, including other trematodes, and also nematode and protozoan parasites such asaquaporin-3 from *Clonorchis sinensis* (GAA31414) [30] (Figure 2). *Ov*-AQP-2 formed a robust clade with aquaporin-9 from *C. sinensis* (GAA55320 and GAA55323) [30] and a group of aquaporins from the schistosomes (blood flukes) [12]. *Ov*-AQP-1 was less closely related to AQPs from other trematodes, including *Ov*-AQP-2, branching at amore distant position in the phylogram along with a predicted AQP from *C. sinensis*, GAA31414). However, both new *Ov*-AQP water channel proteins branched with the aquaglyceroporins rather than the ‘classical’ (strict) aquaporins.

### Developmental expression of *Ov-aqp* genes

**Figure 3:**
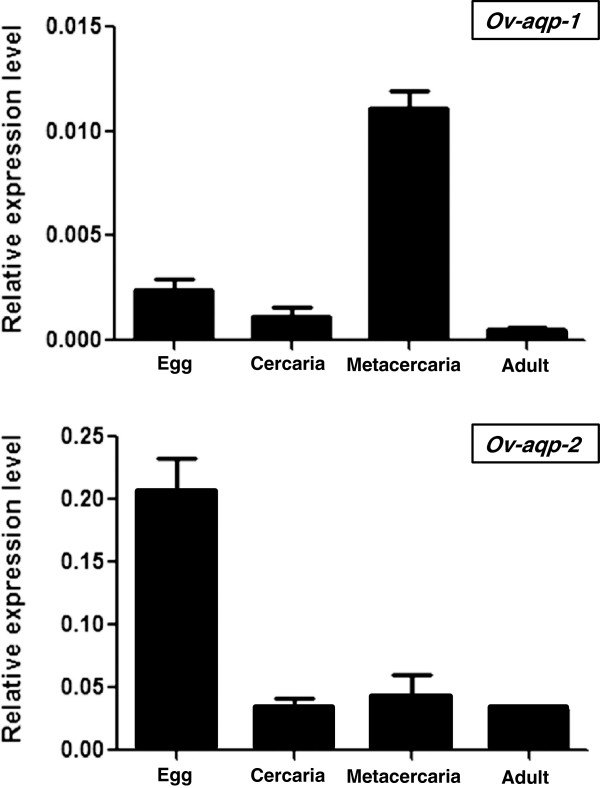
**Levels of expression of mRNAs of *Ov*-*aqp-1 *and *Ov*-*aqp-2 *among developmental stages of the liver fluke*, Opisthorchis viverrini*.** Expression levels relative to *Ov-actin* were analyzed by real-time qRT-PCR. Two biological samples plus technical duplicates were used to assess expression levels. Data are presented as mean ±1SD.

Levels of expression of *Ov-aqp-1 *and -*aqp-2* mRNAs relative to expression of the *actin* control gene were examined by qRT-PCR among developmental stages of *O. viverrini*. Expression of *Ov*-*aqp*-1 expression was seen in all developmental stages examined but was highest in metacercariae (Figure 3). *Ov*-*aqp-2* expression was also detected in all stages; however, highest expression was evident in the egg stage (Figure 3).

### Suppression of *Ov-aqp* gene expression

**Figure 4:**
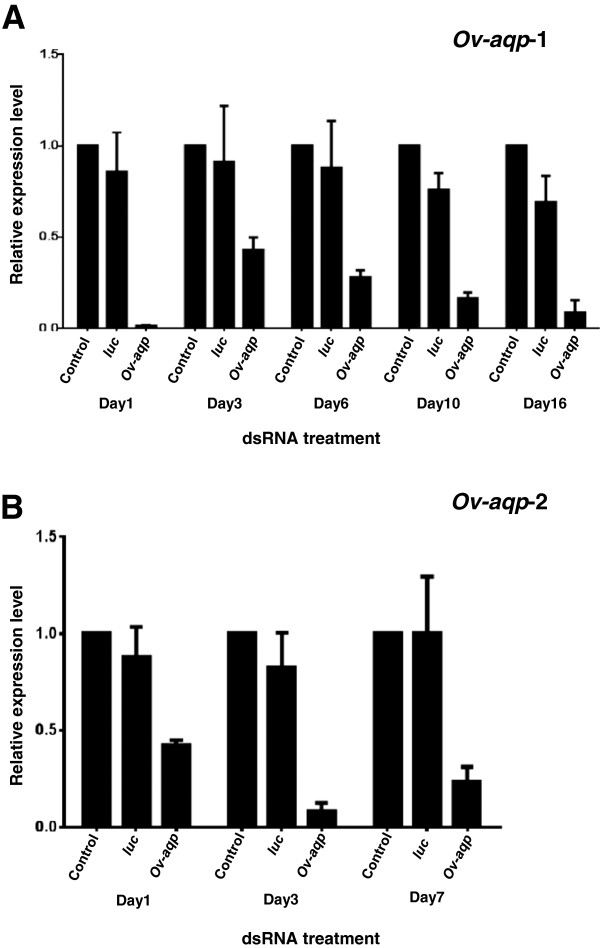
**Suppression of aquaporin *Ov-aqp *mRNA in adult *O. viverrini *by RNA interference (RNAi).** Relative *Ov-aqp-1* and *Ov-aqp-2* expression in adult *Opisthorchis viverrini* flukes following electroporation with dsRNAs corresponding to *Ov-aqp-1***(panel A)** and *Ov*-*aqp-2***(panel B)**. Transcript levels were determined using SYBR-green real time qRT-PCR. *Ov-actin* was used as the control gene for normalization. The control group (no dsRNA treatment) was compared to the negative control group that received *luc* dsRNA. Three biological samples with technical duplicates were used for analysis. Means ±1SD are shown.

*Ov-aqp-1* and *Ov-aqp-2* gene expression was suppressed in *O. viverrini in vitro* by introducing target-specific dsRNAs using electroporation followed by soaking to mediate knockdown. Figure 4A shows the specific suppression of *Ov-aqp-1* by 99%, 57%, 72%, 83% and 91% measured on days 1, 3 6, 10 and 16 after treatment with the dsRNAs, respectively. Figure 4B shows the specific suppression of *Ov-aqp-2* by 58%, 92% and 77% measured on days 1, 3 and 7 after treatment with the dsRNAs, respectively, compared to the negative control group that received *luc* dsRNA.

### Reduced *Ov-*AQP water transport in *Ov-aqp* dsRNA-treated flukes

**Figure 5:**
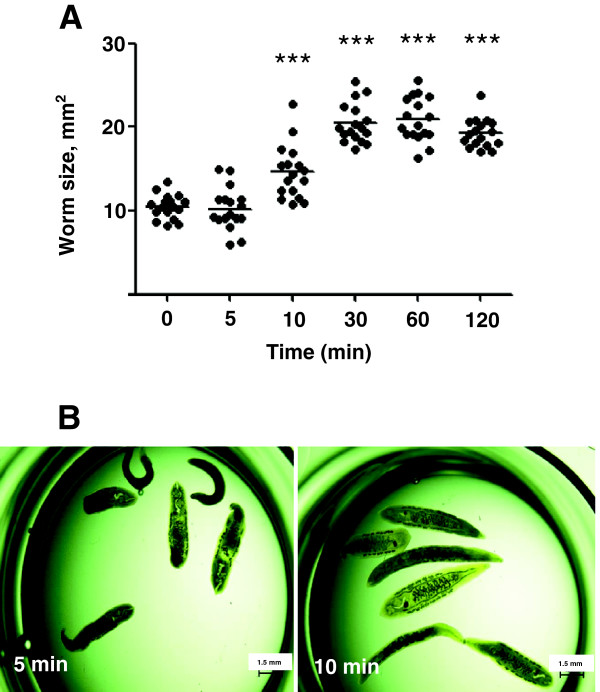
**Swelling of adult *Opisthorchis viverrini *when incubated in distilled water.** Each point represents an individual parasite; the horizontal line indicates the mean for that group; n = 18 per treatment. *p* ≤ 0.001 denoted with *** **(panel A)**. Morphology of a typical set of worms for each group is shown **(panel B)**.

At the outset, to assess the capacity of adult *O.viverrini* to swell following prolonged incubation in distilled water, parasites were measured at intervals after transfer from physiological conditions *in vitro* (i.e. in RPMI) to hypotonic conditions (distilled water). This led to marked swelling of the worms by 10 min, with the increase stabilizing by 30 min of incubation (Figure 5). The average size of worms at time 0 min was 10.5 mm^2^; after 5 min of incubation in water the average size of worms was 10.2 mm^2^ (*p* ≤ 0.001) (Figure 5A). However, after 10 min of incubation in water, average worm size increased to 14.6 mm^2^ (*p* ≤ 0.001) and 20.45 mm^2^ (*p* ≤ 0.001) by 30 min of incubation. After 30 min in water, worms did not continue to swell further for the duration of the experiment (120 min). Based on these findings, further studies were carried out using the 10 min time point.

**Figure 6:**
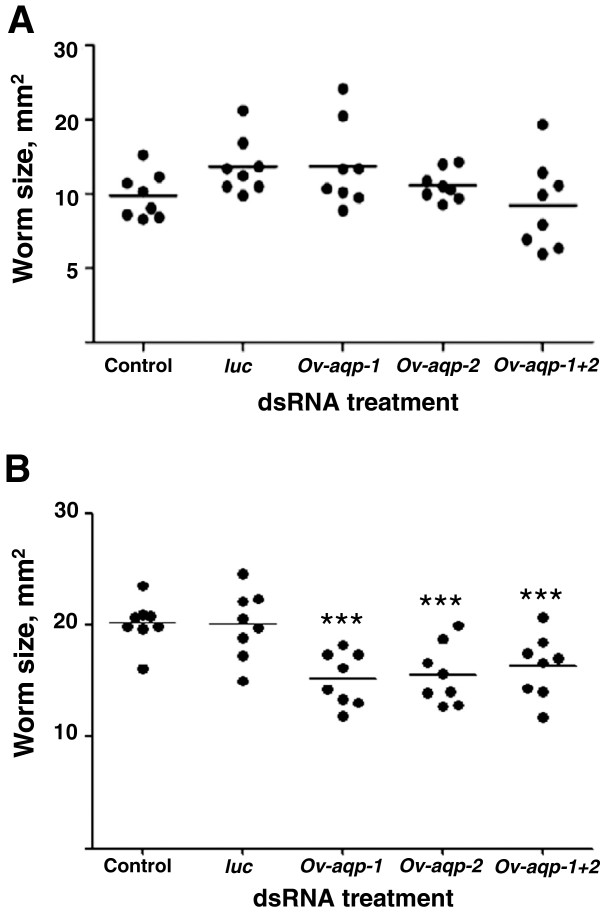
**Suppression of *Ov-aqp-1*, *Ov-aqp-2 *and *Ov-aqp-1 + 2 *gene expression results in an inability to maintain osmotic flux.** Size of cultured adult parasites after treatment with control (*luc*), *Ov-aqp-1*, *Ov-aqp-2* and *Ov-aqp-1 + 2*dsRNAs when incubated in RPMI medium **(A)** or hypotonic medium (distilled water; **B**). Each point represents an individual parasite; horizontal line indicates the mean for that group; *n* = 8 per treatment. *p* ≤ 0.001 is indicated with ***.

To determine whether silencing of *Ov-aqp-1*, *Ov-aqp-2* and *Ov-aqp-1 + 2* expression affected water movement in *O. viverrini*, adult flukes treated with *Ov-aqp* dsRNAs were transferred from RPMI into hypotonic medium as described above (distilled water). Parasites were photographed before and 10 min after transfer into water, and the sizes of a sample of individual parasites from control (mock control or *luc*) and *Ov-aqp*-suppressed groups were compared. When flukes were not subjected to osmotic shock by culture in RPMI medium, no significant difference in body size was detected between any of the control or dsRNA-treated flukes (range = 9.2 – 11.8 mm^2^; Figure 6A). When flukes were transferred into water, control flukes swelled to roughly twice their original size (in RPMI) with average sizes of 20.2 mm^2^ (mock control) and 19.2 mm^2^ (*luc*), however, flukes treated with *Ov-aqp-1*, *Ov-aqp-2* and *Ov-aqp-1 + 2* dsRNAs had significantly reduced average body sizes of15.55 (*p* ≤ 0.001), 16.05 (*p* ≤ 0.001) and 16.3 mm^2^ (*p* ≤ 0.001), respectively (Figure 6B). The mean body sizes of mock and *luc* control groups were not significantly different.

**Figure 7:**
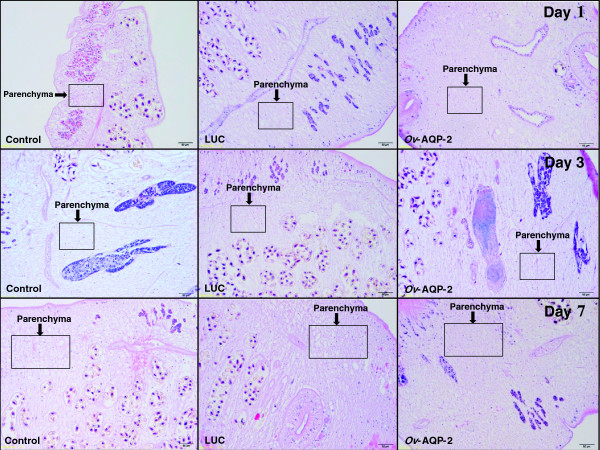
**Cross-section of adult *Opisthorchis viverrini *after RNAi-mediated suppression of *Ov-aqp-2*; stained with hematoxylin and eosin.** Adult *O. viverrini* were electroporated with *Ov*-*aqp*-*2* dsRNA and were maintained thereafter in isotonic medium (RPMI), and observed on days 1, 3 and 7. Regions of trematode parenchyma are indicated by the boxes.

Tissue sections of *Ov-aqp*-*2* dsRNA-treated worms maintained in isotonic culture medium (RPMI) for 7 days showed that the parenchyma was more condensed than control worms (Figure 7), and parenchyma pore sizes were reduced in *Ov-aqp*-*2* dsRNA-treated worms (Figure 7).

## Discussion

*O. viverrini* has a complex life cycle with each developmental stage inhabiting dissimilar environments. Free-living stages including miracidia and cercariae live in fresh water for short periods before invading their respective snail and fish hosts. Parasitic stages including sporocysts, rediae, metacercariae and adult flukes live in the snail (sporocyst, redia), fish (metacercaria) and mammalian (adult fluke) hosts [31]. Like other eukaryotes, trematodes need to regulate water movement into and out of their cells. Transcriptomic and proteomic surveys of *O. viverrini* indicate abundant expression of a small number of AQPs, transporters that have been well characterized as water channels in other species [16,17,22].

In this study, cDNAs encoding *aqp* genes from *O. viverrini* were identified and cloned. AQP is a membrane integral protein that forms pores that facilitate passage of water and other small solutes through cell membranes.They function in cells where the membrane is intact and there is a need for a specific amount of water in the cell to maintain osmotic pressure. The structure of AQPs is well known [10]. *Ov*-AQP-1 and AQP-2 exhibited the typical AQP structure of six transmembrane domains with two NPA boxes that allow pore formation [9,32].

The AQP family is comprised of three groups based on permeability properties. The first group contains the strict AQPs that allow only water to permeate the channel. Members of the second group conduct other small solutes, such as glycerol and urea; these transporters are termed aquaglyceroporins [9,33]. The third family of AQPs has a unique group of conserved residues surrounding the NPA boxes; this group has numerous names including the S-aquaporins (superaquaporins), aquaporins with unusual (or deviated) NPA boxes, and subcellular (or sip-like) aquaporins [11,33,34]. To date, the structures of nematode and trematode AQPs have generally consisted of six transmembrane domains and exhibited two NPA domain-containing hemipores that overlap to form a pore between the membrane bilayer,a defining characteristic of AQPs [13,35]. Some aquaporins from the liver fluke *Fasciola gigantica* do not show conserved two NPA motifs, where the first NPA box is replaced by TAA although the second NPA box is conserved [36]. It is notable, therefore, that the *O.viverrini* AQPs reported here showed higher sequence similarity with the aquaglyceroporin group (human AQP3, -7, -9 and -10) [9,10] as well as *Sm*AQP from the human blood fluke, *S. mansoni *[12] and *Pf*AQP from *Plasmodium falciparum* which exhibit a functional channel facilitating water and glycerol [37] compared to the traditional AQP group (human AQP-1, -2, -5 and -6) [9,10], suggesting that permeants beyond water or glycerol might be transported into/from cells of *O. viverrini*.

Transcripts encoding *aqp* were detected in each of the developmental stages examined here*,* suggesting a requirement for these proteins throughout the entire life cycle of the parasite. The highest expression levels of *Ov-aqp-1* and *Ov*-*aqp-2* were detected in metacercariae and eggs, respectively, implying key roles in the physiology of the infectious process for AQP-1 in particular. To further address the functional roles of *Ov*-AQPs, we used RNAi to suppress their expression and observed that both *Ov-aqp-1* and *aqp-2* were essential for controlling osmosis in the adult fluke. Likewise, *Sm*AQP is important for both entry and exit of water and other solutes in schistosomes [12]. Accordingly, AQP is being considered as a potential vaccine candidate for combating *Schistosoma japonicum* infections due to the presence of numerous B cell epitopes that are predicted to be accessible to host antibodies [38].

Histological analysis of *Ov*-*aqp-2* dsRNA-treated flukes showed that the average pore size in the parenchymal tissue was reduced compared to control worms. This finding, coupled with the inability of *aqp-2-*dsRNA treated worms to maintain osmotic pressure, implies that *Ov-*AQP-2 is integral to entry and egress of water from the tissues of *O. viverrini*. Phylogenetic analysis revealed that the *O. viverrini* AQPs belong to the aquaglyceroporin group, suggesting that their roles extend to substrates beyond other small solutes [37,39,40]. However, in this study we investigated only regulation of water into the body wall. Several authors have investigated other functions of AQPs in schistosomes, including the entry/exit of drugs, highlighting the importance of the trematode tegument in osmoregulation, and the uptake and excretion of metabolic waste and drugs [12,14]. In like fashion, *Ov*-AQPs might play similar roles in drug uptake and waste excretion, and we propose to explore these potential functions in the future. In conclusion, AQPs likely perform essential roles in the movement of water between the external environment and the liver fluke. Drugs that counteract mammalian AQPs are in development [41,42], and accordingly liver fluke AQPs represent potential new targets for interventions to control and treat this carcinogenic pathogen.

## Conclusion

Aquaporins (AQPs) belong to the major intrinsic protein superfamily of integral plasma membrane channel proteins that selectively transport water across cell membranes. AQPs play key roles as water and ion transport channels through the tegument of trematodes. Here we investigated two discrete forms of AQP mRNAs from the adult *O. viverrini*. Phylogenetic analysis indicated that *Ov*-AQPs belong to aquaglyceroporin-like water channel proteins characterized from parasites, which may be integral to the specialized requirements of water and solute control during parasitism. *Ov-aqps* transcripts were detected in all developmental stages of *O. viverrini*. RNA interference using electroporated dsRNA suppressed transcript levels of *Ov-aqps* in adult worms by 58-99%, and disabled water transport in adult flukes.The apparently pivotal roles of *Ov*-AQP in solute homeostasis at the fluke surface suggest that deeper investigation will be informative for the pathophysiology of *O. viverrini*, and may uncover intervention targets, particularly in view of the singularly notable predilection of this pathogen for residence within the biliary tree.

## Competing interests

The authors declare that they have no competing interests.

## Authors’ contributions

ST, PJB, AL and TL conceived and designed the study. ST and SP performed experiments. ST, SK and TL performed data analysis and interpretation. ST, PJB, AL and TL wrote the manuscript. All authors reviewed the draft and accepted it for submission. All authors read and approved the final version of the manuscript.
